# Prospective evaluation of CAR-T cell therapy-related proteinuria and kidney dysfunction

**DOI:** 10.1093/ckj/sfaf270

**Published:** 2025-08-27

**Authors:** Francesc Moncho-Francés, Rafael Hernani, Isabel Juan-García, Ana Benzaquén, Miguel Ángel Solís-Salguero, Ariadna Pérez-Martínez, Juan F Navarro-González, Jose Luis Piñana, Juan Carlos Hernández-Boluda, Maria José Terol Casterá, Carlos Solano, Jose L Górriz, Isidro Torregrosa

**Affiliations:** Nephrology Department, Hospital Clínico Universitario de Valencia, INCLIVA Biomedical Research Institute, Valencia, Spain; Hematology Department, Hospital Clínico Universitario Valencia, INCLIVA Biomedical Research Institute, Valencia, Spain; Nephrology Department, Hospital Clínico Universitario de Valencia, INCLIVA Biomedical Research Institute, Valencia, Spain; Hematology Department, Hospital Clínico Universitario Valencia, INCLIVA Biomedical Research Institute, Valencia, Spain; Nephrology Department, Hospital Clínico Universitario de Valencia, INCLIVA Biomedical Research Institute, Valencia, Spain; Hematology Department, Hospital Clínico Universitario Valencia, INCLIVA Biomedical Research Institute, Valencia, Spain; Research Unit and Nephrology Department, Hospital Universitario Nuestra Señora de Candelaria, Santa Cruz de Tenerife, Spain; Institute of Biomedical Technologies, Universidad de La Laguna, La Laguna, Santa Cruz de Tenerife, Spain; Faculty of Health Sciences, Universidad Fernando Pessoa Canarias, Las Palmas de Gran Canaria, Spain; RICORS2040-RENAL (RD24/0004/0022), Instituto de Salud Carlos III, Madrid, Spain; Hematology Department, Hospital Clínico Universitario Valencia, INCLIVA Biomedical Research Institute, Valencia, Spain; Hematology Department, Hospital Clínico Universitario Valencia, INCLIVA Biomedical Research Institute, Valencia, Spain; Department of Medicine, Faculty of Medicine, Universitat de València, Valencia, Spain; Hematology Department, Hospital Clínico Universitario Valencia, INCLIVA Biomedical Research Institute, Valencia, Spain; Department of Medicine, Faculty of Medicine, Universitat de València, Valencia, Spain; Hematology Department, Hospital Clínico Universitario Valencia, INCLIVA Biomedical Research Institute, Valencia, Spain; Department of Medicine, Faculty of Medicine, Universitat de València, Valencia, Spain; Nephrology Department, Hospital Clínico Universitario de Valencia, INCLIVA Biomedical Research Institute, Valencia, Spain; RICORS2040-RENAL (RD24/0004/0022), Instituto de Salud Carlos III, Madrid, Spain; Department of Medicine, Faculty of Medicine, Universitat de València, Valencia, Spain; Nephrology Department, Hospital Clínico Universitario de Valencia, INCLIVA Biomedical Research Institute, Valencia, Spain; Department of Medicine, Faculty of Medicine, Universitat de València, Valencia, Spain

**Keywords:** acute kidney injury, CAR-T therapy, electrolyte disorders, proteinuria

## Abstract

**Background:**

The nephrotoxicity of chimeric antigen receptor T-cell (CAR-T) therapy has been reported, yet data on urinary abnormalities remain limited. This study aimed to characterize proteinuria, acute kidney injury (AKI), and associated electrolyte disorders in patients treated with CAR-T therapy.

**Methods:**

A prospective analysis was conducted on 63 patients treated with CAR-T therapy between June 2020 and December 2023. Blood tests were collected daily during hospitalization and a first morning urine void was collected on day of infusion (D0), +7 (D7), and +14 (D14).

**Results:**

During the study period, 12 patients (19%) developed AKI. The median urine protein-to-creatinine ratio (uPCR) increased significantly from 0.17 g/g on D0 to 0.39 g/g on D7, returning to baseline levels at D14. Proteinuria >0.5 g/g was detected in 37% of patients at D7. Patients with AKI before D7 had higher proteinuria than patients without AKI or AKI after D7. Proteinuria was associated with higher levels of lactate dehydrogenase (LDH), interleukin-6 levels and cytokine release syndrome (CRS) grade $\ge$3. Patients with higher proteinuria had more progression of hematological disease and increased mortality in the first year. Furthermore, 60 patients (95%) experienced at least one electrolyte disorder, hypophosphatemia being the most frequent. Higher proteinuria was associated with lower levels of phosphorus, potassium, and sodium.

**Conclusions:**

CAR-T therapy is associated with transient renal adverse effects, including proteinuria, AKI, and electrolyte disorders. Systemic inflammation-mediated tubular injury contributed to these adverse effects. These findings enhance our understanding of CAR-T therapy nephrotoxicity. Proteinuria levels at D0 may be associated with disease progression and mortality within the first year.

## GRAPHICAL ABSTRACT

**GRAPHICAL ABSTRACT:**
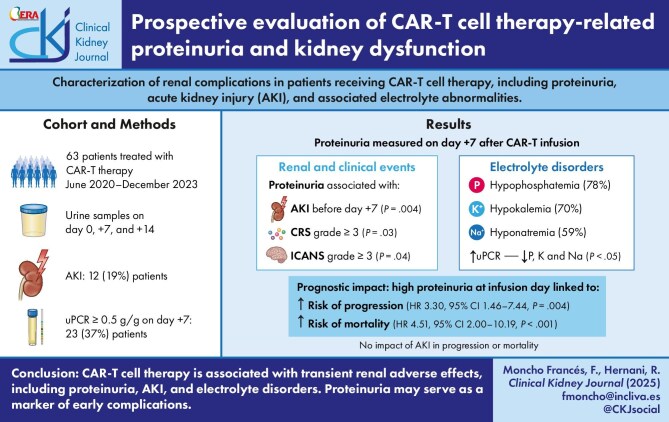

KEY LEARNING POINTS **What was known:**

- CAR-T cell therapy is a novel immunotherapy and its indications are expanding.
- AKI associated with CAR-T therapy is common but mild.
- Urinary abnormalities in these patients have not been described.

**This study adds:**

- Transient proteinuria is common in CAR-T therapy and is associated with tumor burden and systemic inflammation.
- Electrolyte disorders are frequent in CAR-T therapy and their severity is associated with proteinuria.
- AKI within 7 days after CAR-T therapy was associated with higher proteinuria, probably related to systemic inflammation.
- Greater proteinuria may be a marker of increased risk of disease progression and mortality.

**Potential impact:**

- Close monitoring of proteinuria and electrolytes should be considered after CAR-T cell therapy.
- New insights into CAR-T therapy-associated nephrotoxicity.
- Understanding the role of proteinuria in CAR-T therapy could improve early identification of therapy-related adverse events and mortality.

## INTRODUCTION

Chimeric antigen receptor T-cell (CAR-T) therapy has improved the prognosis of patients with refractory/relapsed (R/R) hematological malignancies [1]. Its indications are expanding, and may in the future also include diseases outside the spectrum of malignancies, such as autoimmune diseases [2] and even some infections [3]. Therapy is based on genetically modifying T lymphocytes collected by apheresis to express a chimeric antigen receptor (CAR), and subsequently reinfusing them into the patient, where they can recognize and attack tumor cells [4].

The most common adverse event associated with CAR-T therapy is cytokine release syndrome (CRS), which affects between 42% and 93% of CAR-T patients. CRS is characterized by a febrile syndrome that may progress to hypotension, hypoxia, and severe organ dysfunction. Interleukin-6 (IL-6) plays a central role in its pathogenesis, making IL-6-blocking agents the first-line treatment.

The second most significant adverse event is immune effector cell-associated neurotoxicity syndrome (ICANS), which is less frequent than CRS and typically has a later onset. ICANS manifests with neurological disturbances of varying severity, ranging from mild tremors to seizures. Management involves corticosteroids, with IL-1 receptor antagonists usually reserved for refractory cases [5].

Renal complications are increasingly recognized in CAR-T therapy. Previous retrospective studies have reported an incidence of acute kidney injury (AKI) ranging from 19% to 30%, though data on its severity remain conflicting [6, 7]. Hypophosphatemia is the most frequent and clinically significant electrolyte disturbance, followed by hyponatremia and hypokalemia.

Given the increasing number of indications and patients undergoing CAR-T therapy and its potential nephrotoxicity, this prospective study was conducted to describe AKI and urinary alterations in CAR-T therapy.

## MATERIALS AND METHODS

### Study population

A prospective, single-center study of a case series was conducted including all adult patients with B-cell lymphoma consecutively treated with CAR-T therapy at Hospital Clínico Universitario de Valencia from 1 June 2020 to 31 December 2023. The study included two follow-up phases. During hospitalization, daily routine laboratory tests were collected, and first morning void urine samples were obtained on days 0 (D0), +7 (D7), and +14 (D14). Values below the lower limit of quantification were imputed assuming a normal distribution. After discharge, follow-up was limited to assessing disease progression mortality and creatinine values until 31 December 2024. All the protocols complied with the ethical standards of the Declaration of Helsinki and were reviewed and approved by the Clinical Ethics Committee of the Hospital Clínico Universitario de Valencia, and informed consent was obtained from all patients (reference number 2021/220).

Clinical data were retrieved from the hospital's clinical management information system. These included demographic information, hematologic malignancy type, chemotherapy treatments from diagnosis to CAR-T therapy, hematologic prognosis tools and comorbidities such as diabetes mellitus, hypertension, and chronic kidney disease (CKD).

### Definitions

Baseline creatinine was defined as the creatinine level at the time of apheresis. AKI was defined and classified according to the KDIGO criteria [8] based on the increase in serum creatinine levels. Hyponatremia was defined as a plasma sodium level <135 mmol/l (mild 130–135 mmol/l, moderate 125–130 mmol/l, severe <125 mmol/l). Hypokalemia was defined as potassium levels < 3.5 mmol/l (mild 3–3.5 mmol/l, moderate 2.5–3 mmol/l, severe <2.5 mmol/l), and hypophosphatemia as phosphorus levels <2.5 mg/dl (mild 2–2.5 mg/dl, moderate 1–2 mg/dl, severe <1 mg/dl). Hypomagnesemia was defined as magnesium levels <1.7 mg/dl and hypocalcemia as calcium levels <8.5 mg/dl.

CRS and ICANS were graded according to American Society for Transplantation and Cellular Therapy recommendations [9] and managed following institutional guidelines [10]. Severe forms of CRS or ICANS were defined as grade 3 or higher.

Overall response rate was defined as the proportion of patients who achieved either partial (PR) or complete response (CR) after CAR-T infusion [11]. Progression-free survival (PFS) was defined as the time from CAR-T infusion until relapse, progression, or death from any cause. Disease status before CAR-T therapy was defined as: (i) primary refractory, in patients who never achieved end of treatment response, or (ii) prior chemosensitivity, in patients who responded to previous treatment but experienced R/R before CAR-T. The Endothelial Activation and Stress Index (EASIX) score was calculated prior to lymphodepleting chemotherapy, as previously described [12]. The International Prognostic Index (IPI) was calculated based on five clinical factors: age > 60 years, stage III/IV disease, elevated LDH, ECOG performance status ≥2, and >1 extranodal site [13].

### Statistical analysis

Statistical analysis was conducted using SPSS statistics (v.25) and RStudio (The CRAN Project). Categorical variables were expressed as numbers and percentages, and continuous variables as mean $\pm$ SD or median with interquartile range (IQR). The normal distribution assumption was assessed with the Shapiro–Wilk test. Between-group differences were evaluated using the Student's *t*-test or the Mann–Whitney *U*-test for quantitative variables, and the Chi-square test or Fisher's exact test for qualitative variables. Multivariable logistic regression was performed to assess risk factors and the Kaplan–Meier estimator and Cox-proportional hazards model to compare mortality and progression between groups. A *P* value <.05 was considered statistically significant.

For subgroup analyses, patients were stratified according to a uPCR threshold of 0.5 g/g. This cutoff was selected based on internal data: ROC analysis and the upper tertile of uPCR at D7.

## RESULTS

### Patient characteristics

CAR-T was administered to 63 patients during the study period. Three different types of CAR-T constructs were infused: axicabtagene ciloleucel (axi-cel, *n* = 47; 75%), tisagenlecleucel (tisa-cel, *n* = 10, 16%), and 4-1BB investigational product (4IP, *n* = 6, 9%). Baseline characteristics are summarized in Table 1. Briefly, 90% of patients had diffuse large B-cell lymphoma and all patients were R/R after two lines of systemic therapy. Although metabolic tumor volume was not available, we included other surrogate markers of tumor burden, such as EASIX score and LDH levels. Fifty-nine (94%) had received at least one platin-based schema. Fludarabine in combination with cyclophosphamide was the most frequent regimen used for lymphodepletion (*n* = 62, 98%). Seven patients required dose adjustment for lymphodepletion because of CKD.

**Table 1:** Baseline characteristics of patients receiving CAR-T therapy according to uPCR at D7.

		Urine protein-to-creatinine ratio, g/g		
	*N* = 61	$<$ 0.5 (*n* = 38)	≥0.5 (*n* = 23)	*P*
Age in years, median (IQR)	62 (49–68)	62 (49–68)	62 (49–70)	1
Male sex, *n* (%)	34 (55)	25 (66)	9 (39)	**.04**
Serum creatinine at apheresis in mg/dl, median (IQR)	0.83 (0.64–0.96)	0.86 (0.67–0.97)	0.72 (0.60–0.95)	.33
Serum creatinine at infusion in mg/dl, median (IQR)	0.70 (0.51–0.87)	0.73 (0.53–0.89)	0.62 (0.49–0.84)	.32
Hypertension, *n* (%)	18 (28)	12 (32)	6 (26)	.65
Diabetes, *n* (%)	9 (14)	6 (16)	3 (13)	1
CKD, *n* (%)	7 (11)	4 (10)	3 (13)	1
LDH preLD in U/l, median (IQR)	548 (366–1146)	443 (285–606)	966 (541–1994)	**<.001**
EASIX score preLD, median (IQR)	2.67 (1.51–6.76)	2.09 (1.17–4.40)	5.47 (3.44–11.98)	**.001**
Diagnosis, *n* (%)				1
DLBCL	56 (92)	35 (92)	21 (93)	
PMBCL	5 (8)	3 (8)	2 (7)	
IPI preLD $\ge$3, *n* (%)	31 (51)	17 (45)	14 (61)	.22
Ann Arbor stage preLD ${\mathrm{III}} - {\mathrm{IV}}$, *n* (%)	40 (68)	22 (61)	18 (78)	.17
Disease status preLD, *n* (%)^a^				**.02**
Disease progression	40 (69)	19 (55)	21 (92)	
Stable disease	6 (10)	5 (14)	1 (4)	
Partial response	6 (10)	5 (14)	1 (4)	
Complete response	6 (10)	6 (17)	0 (0)	
Primary refractory, *n* (%)	34 (56)	22 (59)	12 (52)	.58
Prior autologous HSTC, *n* (%)	10 (16)	7 (18)	3 (13)	.72
CAR-T cell construct, *n* (%)				.14
Tisagenlecleucel	10 (16)	7 (18)	3 (13)	
Axicabtagene ciloleucel	46 (75)	26 (68)	20 (87)	
Investigational product 4-1BB,	5 (8)	5 (13)	0 (0)	

DLBCL, diffuse large B-cell lymphoma; PMBCL, primary mediastinal B-cell lymphoma; preLD: pre-lymphodepletion; HSTC, hematopoietic stem-cell transplantation.

^a^Disease status at LD was not assessed in three patients.

Bold text indicates statistically significant results (P < .05).

### Proteinuria

A first morning void urine sample was collected from 62 (98%) patients on D0, 61 (96%) on D7, and 58 (92%) on D14. The median urine protein-to-creatinine ratio (uPCR) was 0.17 g/g (IQR, 0.09–0.31) on D0, 0.39 g/g (IQR, 0.17–0.83) on D7, and 0.20 g/g (IQR, 0.11–0.37) on D14. There was a significant difference in uPCR between D0 and D7 (*P* = .003), but not between D0 and D14 (*P* = .12). Eleven (17%) patients had proteinuria higher than 0.5 g/g at D0, 23 (37%) at D7 and 12 (19%) at D14. Patients were stratified according to their uPCR level at D7 (<0.5 versus $\ge$0.5 g/g) to compare their baseline characteristics (Table 1). Patients with greater tumor burden had higher levels of proteinuria at D7.

**Figure 1:**
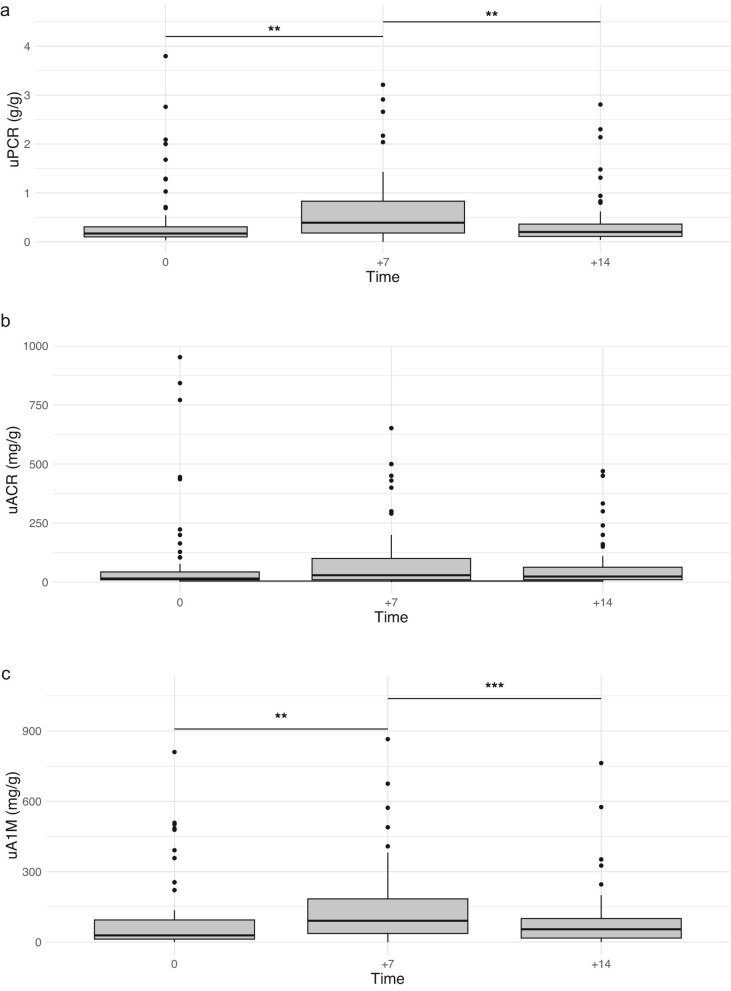
Changes in proteinuria during CAR-T therapy. (**a**) uPCR was significantly increased at day 7 post-CAR-T therapy, indicating transient proteinuria, with levels returning closer to baseline by day 14. (**b**) uACR showed a moderate rise at day 7 post-therapy, reflecting temporary albuminuria, but was largely stabilized by day 14. (**c**) uA1M levels were significantly elevated at day 7, suggesting tubular proteinuria, with a partial reduction by day 14.

The median urinary albumin-to-creatinine ratio (uACR) was 15 mg/g (IQR, 9–44) on D0, 29 mg/g (IQR, 10–100) on D7, and 23 mg/g (IQR, 10–65) on D14. The median urinary alpha-1-microglobulin-to-creatinine ratio (uA1M) was 28 mg/g (IQR, 12–96) on D0, 90 mg/g (IQR, 35–186) on D7, and 54 mg/g (IQR, 16–100) on D14. There was a significant difference between D0 and D7 in uA1M (*P* = .004), but not in uACR. No patients had a uACR/uPCR ratio >50% (Fig. 1).

### Acute kidney injury

During the follow-up period, 12 patients (19%) developed AKI. The median time from CAR-T infusion to the peak serum creatinine value was 5 days (IQR 4–11).

According to the KDIGO criteria, nine patients presented with AKI stage 1, one patient AKI stage 2, and two patients AKI stage 3. No patients required renal replacement therapy. The mean peak serum creatinine in patients with AKI was 1.54 ± 0.48 mg/dl.

Baseline characteristics were compared between groups based on the presence or absence of AKI, without identifying any risk factors (Supplemental Material, Table S1). Nine patients developed AKI before D7, compared with three patients after D7. In patients with AKI before D7, there were no differences between basal creatinine levels and creatinine levels at D14 [0.89 mg/dl (IQR, 0.51–1.2) vs 0.90 mg/dl (IQR, 0.47–1.36), *P* = .610].

Patients who presented AKI before D7 had greater levels of uPCR than patients without AKI or with AKI after D7 [1.0 g/g (IQR, 0.5*–*2.1) vs. 0.29 g/g (IQR, 0.13–0.65), *P* = .004] (Supplemental material, Fig. S1).

### Electrolyte disorders

**Figure 2:**
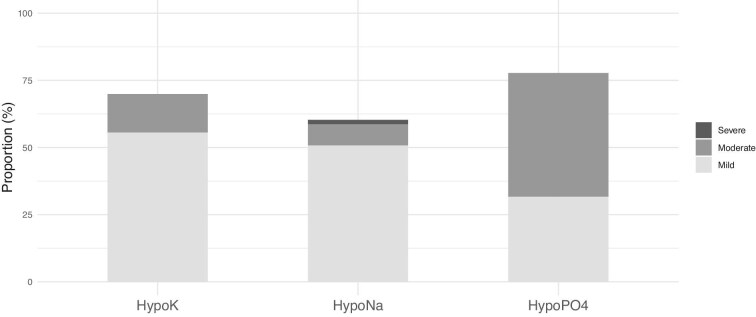
Proportions of electrolyte disturbances (HypoK, HypoNa, HypoPO4) categorized by severity after CAR-T therapy.

Sixty (95%) patients experienced at least one mild electrolyte disorder, 30 (51%) patients had a moderate disorder, and one (2%) patient experienced a severe disorder. Twenty-five (40%) patients had hyponatremia, hypokalemia, and hypophosphatemia, while 21(33%) patients had two different electrolyte disorders. The most common disorder was hypophosphatemia (78%), in terms of both severity and total number of patients affected, followed by hypokalemia (70%) and hyponatremia (59%) (Fig. 2). Other electrolyte disorders included hypomagnesemia in 35 (56%) patients and hypocalcemia in 9 (15%) patients. The median time from infusion to nadir for electrolyte disorder was five days (IQR, 4–8) after CAR-T infusion.

Patients with higher uPCR at D7 exhibited lower nadir levels of sodium (*r* = −0.42, *P* = .001), potassium (*r* = −0.38, *P* = .002), and phosphate (*r* = −0.55, *P* < .001).

### CAR-T therapy-related complications

In total, 53 (87%) and 32 (52%) patients developed CRS and ICANS, respectively, with grade 3 or higher CRS and ICANS occurring in 6 (10%) and 15 (26%) patients, respectively. The median time from CAR-T infusion to CRS and ICANS was 1 day (IQR, 1–3) and 7 days (5–8), respectively. Nineteen patients (31%) were admitted to the intensive care unit (ICU). Patients with immunotoxicities were more likely to develop proteinuria at D7 (Table 2). ICANS grade $\ge$3 and higher IL-6 levels were related with the development of AKI before D7 (Supplemental Material, Table S2). For the treatment of these complications, 36 (57%) patients received dexamethasone, and 19 (30%) patients received tocilizumab.

**Table 2:** Adverse events of CAR-T therapy according to uPCR at D7.

		Urine protein-to-creatinine ratio, g/g		
	*N* = 61	$<$ 0.5 (*n* = 38)	$\ge\$ 0.5 (*n* = 23)	*P*
CRS, *n* (%)	53 (87)	31 (82)	22 (96)	.24
CRS grade $\ge$3, *n* (%)	6 (10)	1 (3)	5 (22)	**.03**
ICANS, *n* (%)	32 (52)	16 (42)	16 (70)	**.04**
ICANS grade $\ge$3, *n* (%)	15 (26)	7 (18)	8 (35)	.15
IL-6 peak in pg/ml, median (IQR)	239 (58–1439)	65 (49–235)	1688 (546–5225)	**<.001**
ICU admission, *n* (%)	19 (31)	8 (21)	11 (48)	**.03**
Hospital stays in days, median (IQR)	22 (20–28)	21(19–24)	27 (21–42)	**.005**

Bold text indicates statistically significant results (P < .05).

### Risk factors for proteinuria

The severity of proteinuria at D0 was correlated with higher pre-lymphodepletion LDH levels (*r* = 0.77, *P* < .001), increased IL-6 levels at D0 (*r* = 0.53, *P* < .001), increased C-reactive protein (CRP) at D0 (*r* = 0.57, *P* < .001), and greater pre-lymphodepletion EASIX score (*r* = 0.60, *P* < .001).

The severity of proteinuria at D7 was correlated with higher lactate dehydrogenase (LDH) pre-lymphodepletion (*r* = 0.53, *P* < .001), EASIX (*r* = 0.49, *P* < .001), peak IL-6 levels (*r* = 0.62, *P* < .001), peak CRP levels (*r* = 0.59, *P* < .001), higher degree of CRS (*r* = 0.45, *P* ≤ .001) and higher degree of ICANS (*r* = 0.42, *P* < .001). In multivariate analysis, pre-lymphodepletion LDH levels (*P* = .025), peak IL-6 levels (*P* = .014), and CRS severity (*P* = .002) were assessed as independent predictors for higher proteinuria after CAR-T infusion.

### Response, progression and survival

Forty-six (73%) patients responded [CR, *n* = 35 (56%); PR, *n* = 11 (17%)] at a median follow-up of 359 days (IQR, 120–479). During the follow-up period, 34 (54%) experienced disease progression [refractory, *n* = 15 (24%), relapse, *n* = 19 (30%)]. The median PFS was 242 days (IQR, 56–503). Thirty-five (55.6%) patients died at a median follow-up of 153 days (IQR, 91–284). Causes of death were as follows: R/R (*n* = 28, 80%), infection (*n* = 5, 14%), and ICANS (*n* = 2, 6%).

**Figure 3:**
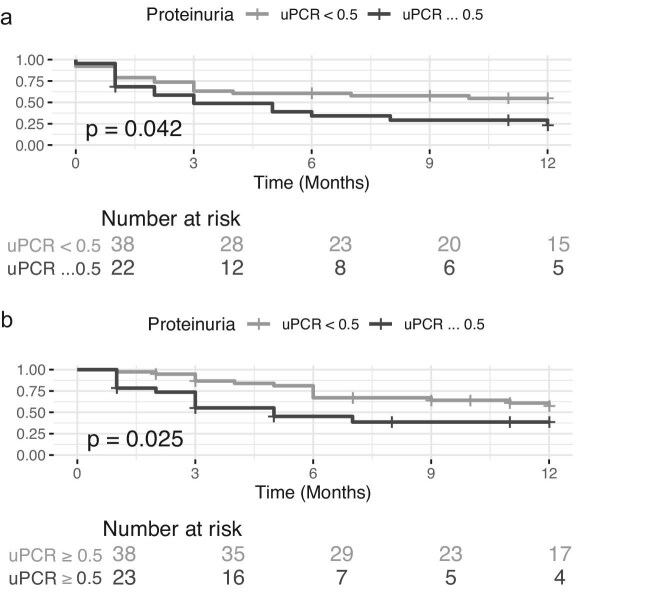
Survival analysis of patients stratified by proteinuria level at day 0. (**a**) Patients with higher proteinuria had a greater risk of hematological disease progression during the first year: log-rank test *P* value. (**b**) Patients with higher proteinuria had a greater risk of death during the first year after CAR-T therapy: log-rank test *P* value.

Univariate analysis showed that proteinuria higher than 0.5 g/g at D0 correlated with a higher risk of progression (HR 3.30, 95% CI 1.46–7.44, *P* = .004) and mortality (HR 4.51, 95% CI 2.00–10.19, *P* < .001) (Fig. 3), whereas AKI had no impact. In multivariate analysis, the presence of elevated proteinuria was significantly associated with an increased risk of death (HR = 5.75, 95% CI 1.43–22.14, *P* = .014), after adjusting for age, CKD, IPI score, hematologic status, LDH, EASIX, and disease stage.

## DISCUSSION

Our findings highlight the transient nature of proteinuria in CAR-T therapy, likely driven by systemic inflammation and tubular injury. The correlation between proteinuria, CRS severity, and IL-6 levels supports an inflammatory-mediated mechanism. In addition, early AKI appears to be inflammation-driven, whereas later AKI may be influenced by secondary factors such as infections or nephrotoxic exposures. Electrolyte disorders, particularly hypophosphatemia, further suggest tubular dysfunction.

Despite the expanding use of CAR-T therapy, its associated nephrotoxicity remains poorly characterized. In this study, we aimed to address this gap by analyzing the incidence of AKI, electrolyte disorders, and proteinuria in patients receiving CAR-T cell therapy. To the best of our knowledge, this is the first study to prospectively analyze proteinuria in this subset of patients.

In our study, 12 patients (19%) developed AKI, a finding consistent with prior research on AKI in CAR-T therapy. Leon-Roman *et al.* reported AKI in 24 out of 115 patients (21%), while Vincedeau *et al.* described AKI in 41 out of 119 patients (35%) [14, 15]. Furthermore, a recent review analyzing nephrotoxicity in CAR-T therapy compiled data from 14 clinical studies and estimated an AKI incidence of 17.3% (95% CI 11.47–23.88), similar to our study [16].

Transient proteinuria was a common finding after CAR-T cell therapy in our study, with a significant increase in uPCR at D7 and partial return to baseline levels at D14. We found that patients presenting AKI before D7 had greater levels of uPCR than patients without AKI or those with AKI after D7. The higher levels of uA1M combined with the absence of significant albuminuria (no patients with uACR/uPCR ratio >0.5) suggest that proteinuria stems predominantly from tubular injury. This phenomenon appears to be associated with markers of higher tumor burden, such as pre-lymphodepletion LDH levels, and of systemic inflammation, including peak IL-6 levels and CRS severity. The inflammatory cascade driven by cytokine release and immune activation—key features of CRS—may play a central role in the endothelial and tubular damage observed in CAR-T patients leading to tubular proteinuria [17]. Interestingly, the only previous trial to analyze proteinuria in this context did not find any relationship between proteinuria and AKI, a difference from our results that could be attributed to our prospective method of urine collection [14].

Presence of elevated proteinuria on the day of CAR-T infusion (uPCR ≥ 0.5 g/g) was associated with a significantly increased risk of mortality (HR = 5.75, 95% CI: 1.43–23.14, *P* = .014), even after adjusting for age, inflammatory markers, disease stage, and prognostic scales. This finding suggests that baseline proteinuria may be a marker of endothelial injury or systemic inflammation present prior to CAR-T administration, which may be linked to the development of early complications of the therapy, as evidenced by the rapid decline in survival observed during the first 3 months. Given the limited number of events, the inclusion of multiple covariates may have introduced a risk of overfitting. However, this analysis may serve as a starting point for future investigations in larger cohorts, which could validate these findings.

No risk factors for AKI were identified in our study; however, we observed an association between patients with AKI before D7 and high IL-6 levels or ICANS $\ge$ grade 3. These findings, together with higher proteinuria levels, led us to hypothesize the existence of two types of AKI in CAR-T therapy: (i) in the first week, AKI could be associated with systemic inflammation and immune activation producing indirect kidney injury mediated by a reduction in intravascular volume and direct tubular injury, leading to the death of tubular cells and subsequent proteinuria; and (ii) after D7, AKI could be associated with indirect CAR-T therapy complications such as sepsis, nephrotoxic medications, and contrast exposure [16].

Electrolyte disorders were frequently observed in CAR-T therapy, although most cases were mild, consistent with findings from Farooqui *et al.* [18] and Gupta *et al.* [7]. Hypophosphatemia emerged as the most important electrolyte disturbance, likely driven by multiple factors, including renal tubular injury mediated by inflammation, increased metabolic demands of CAR-T cells, and possibly the phosphaturic effects of fibroblast growth factor 23 [19]. The association we observed between proteinuria and the severity of electrolyte disturbances strongly supports the hypothesis that tubular injury contributes significantly to electrolyte imbalances during CAR-T therapy [7, 18, 19].

Our study has certain limitations, including the single-center study design, the use of different CAR-T cell constructs, the limited sample size, and focus on early renal complication without long-term follow-up of creatinine or urine abnormalities. Furthermore, the absence of daily urine samples may have led us to underestimate the presence of proteinuria. In addition, we only included uA1M as a surrogate marker of tubular injury, without measuring other specific biomarkers such as Kidney Injury Molecule-1 [20] or Neutrophil Gelatinase-Associated Lipocalin [21], which could have provided deeper insights into the pathophysiology of tubular damage in this setting. Furthermore, the absence of systematic data on supportive care interventions (e.g. intravenous fluids, vasopressors, nephrotoxic drugs) is a limitation, as these therapies may have influenced the incidence and severity of renal adverse events. Nonetheless, the prospective design allows us to provide novel insights into urine findings and less frequent electrolyte disorders such as hypophosphatemia and hypomagnesemia. This approach offers a more comprehensive analysis of CAR-T cell nephrotoxicity.

## CONCLUSION

CAR-T cell therapy is associated with transient renal adverse effects, including proteinuria, AKI, and electrolyte disorders. Proteinuria may serve as a surrogate marker of disease progression and mortality. The findings of this study emphasize systemic inflammation and tubular injury as contributors to these adverse effects and enhance our understanding of CAR-T cell therapy nephrotoxicity.

## Supplementary Material

sfaf270_Supplemental_Files

## Data Availability

The data underlying this article are fully available within the published paper and the supplementary material.
