# Supplementary material for: Prospective evaluation of CAR-T cell therapy-related proteinuria and kidney dysfunction
Source: Clin Kidney J. 2025 Aug 27;18(10):sfaf270. doi: 10.1093/ckj/sfaf270 (PMC12498094; doi:10.1093/ckj/sfaf270)
Supplement: sfaf270_Supplemental_Files [file sfaf270_supplemental_files.zip › SUPPLEMENTARY MATERIAL.docx]

SUPPLEMENTARY MATERIAL

Table S1. Baseline characteristics of patients receiving CAR-T therapy according to the development of AKI .

|  | N = 63 | AKI (n= 12) | No AKI (n =51) | p-value |
| --- | --- | --- | --- | --- |
| Age in years, median (IQR) | 62 (48-68) | 57 (42-70) | 62 (49-67) | 0.67 |
| Male sex, n (%) | 35 (55) | 8 (67) | 27 (53) | 0.38 |
| Serum creatinine at apheresis in mg/dL, median (IQR) | 0,84 (0.65-0.96) | 0.81 (0.61-1.00) | 0.80 (0.85-0.95) | 0.79 |
| Serum creatinine at infusion in mg/dL, median (IQR) | 0.70 (0,50-0.87) | 0.84 (0.71-1.02) | 0.63 (0.51-0.79) | 0.07 |
| Hypertension, n (%) | 18 (28) | 4 (33) | 14 (27) | 0.72 |
| Diabetes, n (%) | 9 (14) | 1 (8) | 8 (16) | 1 |
| CKD, n (%) | 7 (11) | 1 (8) | 6 (11) | 1 |
| LDH preLD in U/L , median (IQR) | 548 (355-1088) | 429 (341-658) | 555 (377-1205) | 0.31 |
| EASIX score pre-LD, median (IQR) | 2.54 (1.48-6.40) | 2.23 (1.34-7.93) | 2.67 (1.48-6.40) | 0.73 |
| Diagnosis, n (%) |  |  |  | 0.32 |
| DLBCL | 57 (91) | 10 (83) | 47 (92) |  |
| PMBCL | 6 (9) | 2 (17) | 4 (8) |  |
| IPI preLD $\geq$ 3, n (%) | 31 (49) | 4 (33) | 27 (53) | 0.22 |
| Ann Arbor stage pre LD $\geq$ 3, n (%) | 41 (65) | 6 (50) | 35 (68) | 0.17 |
| Disease status preLD, n (%) * |  |  |  | 0.46 |
| Disease progression | 41 (68) | 8 (67) | 33 (69) |  |
| Stable disease | 6 (10) | 2 (17) | 4 (8) |  |
| Partial response | 7 (12) | 2 (17) | 5 (10) |  |
| Complete response | 6 (10) | 0 (0) | 6 (13) |  |
| Primary refractory, n (%) | 35 (57) | 6 (50) | 29 (59) | 0.56 |
| Previous Autologous HSTC, n (%) | 10 (16) | 1 (8) | 9 (18) | 0.67 |
| CAR-T cell construct, n (%) |  |  |  | 0.43 |
| Tisgenlecleucel | 10 (15.8) | 5 (21.7) | 5 (12.5) |  |
| Axicabtagene ciloleucel | 47 (74.6) | 15 (65.2) | 32 (80) |  |
| Investigational product 4-1BB | 6 (9.5) | 3 (13.0) | 3 (7.5) |  |

CKD, chronic kidney disease; LDH, lactate dehydrogenase; preLD: prelymphodepletion EASIX, endothelial activation and stress index; DLBCL, diffuse large B-cell lymphoma; PMBCL, primary mediastinal B-cell lymphoma; IPI, international prognostic index; HSTC, hematopoietic stem-cell transplantation, *Disease status preLD was not assessed in 3 patients.

TABLE S2. Adverse events of CAR-T therapy stratified by the presence of AKI before day 7.

|  | N = 63 | AKI (n= 9) | | No AKI (n =54) | p-value |
| --- | --- | --- | --- | --- | --- |
| CRS, n (%) | 54 (86) | 9 (100) | 45 (83) | | 0.11 |
| CRS grade $\geq$ 3, n (%) | 6 (9) | 2 (22) | 4 (7) | | 0.20 |
| ICANS, n (%) | 33 (52) | 6 (67) | 27 (50) | | 0.47 |
| ICANS grade $\geq$ 3, n (%) | 16 (25) | 5 (56) | 11 (20) | | **0.04** |
| IL-6 peaks in pg/mL, median (IQR) | 239 (58-1439) | 1886 (300-17586) | 212 (55-825) | | **0.03** |
| ICU admission, n (%) | 20 (32) | 5 (56) | 15 (28) | | 0.13 |
| Hospital stays in days, median (IQR) | 22 (20-28) | 21(20-26) | 22 (20-28) | | 0.53 |

CRS, cytokine release syndrome; ICANS, immune effector cell-associated neurotoxicity syndrome; IL-6, interleukin 6; ICU, intensive care unit

FIGURE S1. Changes in proteinuria during CART therapy.

Figure S1A. Patients who developed AKI within the first 7 days post-CAR-T therapy exhibited significantly higher uPCR levels at **day +7** compared to those without AKI or those who developed AKI after day 7

Figure S1B. Patients who developed AKI within the first 7 days showed a significant increase in uACR at day +7, compared to those without AKI or with late-onset AKI

Figure S1C. Patients with AKI within the first 7 days exhibited a significant increase in uA1M at day +7 compared to those without AKI or with late-onset AKI,
